# The Making of Clandestinity: Strategic Ignorance in Abortion Practices in Latin America

**DOI:** 10.1080/14616742.2024.2335643

**Published:** 2024-04-22

**Authors:** Cordelia Freeman, Sandra Rodríguez

**Affiliations:** Department of Geography, University of Exeter. Amory Building, Rennes Drive, Exeter, EX4 4RJ; Pontificia Universidad Católica del Perú

**Keywords:** Strategic ignorance, ignorance studies, abortion, reproductive justice, Latin America

## Abstract

Abortion is a public secret in Latin America. It is highly restricted across the majority of the continent and yet millions of abortions take place every year. We use the sociological framework of ‘strategic ignorance’ to argue that convenient not knowing, erasure and concealment allow for the simultaneous negation and allowance of abortions in Latin America. By drawing on interviews with people involved in abortion activism and access across the continent we examine three sets of actors: the state, abortion providers and individuals. When wielded by the state, strategic ignorance reproduces the status quo of the criminalization of abortion but when wielded by abortion providers and individuals it creates the conditions for ‘clandestine’ abortions to be procured without prosecution. Strategic ignorance is therefore mobilized by the powerful as well as the powerless who are resisting state control of their fertility and reproductive lives.

## Introduction

In the vast majority of Latin America abortion is illegal and stigmatized. But abortions are highly common, 6.5 million abortions take place across the region every year (Bearak et al. 2020). We set out to examine this apparent paradox and investigate what maintains this situation where abortion is both allowed and suppressed in Latin America. In this paper we explore how a range of actors strategically ignore, stay silent, conceal, and erase information in ways that allow for abortion access in legally restrictive regimes in Latin America.

Abortion is paradoxical in that it is criminalized and shrouded in stigma procedure while also being a highly common event (Duarte, Silva and Pinto 2020; Roth 2020). The term ‘clandestine’ is prefixed to ‘abortion’ “to evoke what is hidden, what is concealed, what happens in the shadows but is not necessarily completely invisible” (Sutton 2017, 889). Clandestine abortions are not by definition unsafe, but they are more likely to be unsafe and it is low-income, rural, racialized groups who are most likely to experience unsafe abortions and to be prosecuted for accessing them. The situation of abortion in most of Latin America is that it is an ‘open secret’ where even when it is strictly prohibited, many people know how to access one (Kimball 2020; Céspedes, Rentería and Pinto 2020). This builds on Shepard’s (2000,111) idea of the “‘double discourse system,’ which maintains the status quo in repressive or negligent public policies while expanding private sexual and reproductive choices behind the scenes.” This system results in a breach between official, public discourse and what actually occurs in practice, which means that abortion is more commonplace and more accessible than state policy would make it seem. Abortion is a public secret and the oxymoronic nature of this is reproduced through strategic ignorance.

This paper draws on a range of interviews with people involved in abortion activism or access in Latin America. This consists of interviews with 32 participants in Peru and 16 in Mexico. While we are unable to delve into all the complexities of abortion access in both contexts, broadly speaking, abortion is highly restricted in Peru as it is classed as a crime. ‘Therapeutic abortions’ have been legal in theory since 1924 for specific circumstances, but in reality this pathway is inaccessible (Duffy, Freeman and Rodríguez 2023). In Mexico, abortion is legislated on a state-by-state basis, and in 2021, the Supreme Court ruled that the criminalization of abortion was unconstitutional (Herrera García 2022). This decriminalization does not create pathways to access, however, and formidable barriers to abortion access remain. Regardless of the legal status of abortion, many thousands of abortions take place in both Peru and Mexico every year (Bearak et al. 2020; Ferrando 2006; Singer 2022). Therefore, these two countries can act as snapshots of how abortions are accessed when the voluntary termination of pregnancy is illegal or restricted.

The interviews were conducted between January 2020 and November 2022 and the participants were selected through purposive sampling, chosen for their involvement in abortion, and included abortions activists, providers and people involved with organisations from the multinational to the local level, including state actors. These categories often overlapped with people working in state jobs but also identifying as activists. We attempted to interviewee participants from varied regions of each country so in Peru interviewees were based in Lima, Ayacucho, Cajamarca, Arequipa, and Cusco, and in Mexico were in Mexico City, Yucatán, Nevo León, and Baja California. The interviews were wide-ranging and unstructured but centred around abortion law, provision and access. The interview data were anonymized, transcribed and manually coded using inductive coding independently by the authors and then compared. Any quotes used here were translated from the original Spanish by the authors. Ethical approval was granted by the University of Exeter.

In this paper, we show how strategic ignorance upholds the current landscape of abortion in Latin America. On the one hand it keeps abortion an illegal, clandestine act while on the other hand it provides the conditions where clandestine abortions can be sought and kept secret. Leslie Reagan (2022,1) uses the term ‘triangle of interactions’ in her work on abortion to refer to the relationships between the medical profession, state agents and women. We adapt this triangle to focus on three actors: *the state* as the government that controls abortion law and official practice, *providers* who are healthcare professionals, activists and information providers, and *individuals* who are the people accessing abortions. Diverse strategies of strategic ignorance are wielded by state authorities and also by providers and individuals that result in what Ismail (2006, xxxv) calls “the mutual ensnarement of rulers and ruled”. They work in tandem; the latter is a response to the former, but this doesn’t mean that the relation is unilinear. In this paper we set out the seemingly dissimilar strategies of ignorance that together uphold the simultaneous negation and allowance of abortions through an ambivalence that creates the scenario where ‘clandestine’ abortions continue to occur. We begin by providing an overview of the most relevant work on ignorance studies and strategic ignorance, and then explore how three sets of actors, the state, providers and individuals, all enable strategic ignorance about abortion practices in Latin America.

## Locating Strategic Ignorance

Strategic ignorance is an idea developed by sociologists and other scholars and broadly lies within ‘the sociology of ignorance’. This body of scholarship emerged in response to work on the sociology of knowledge as scholars argued that it is just as important to study what we do not know as well as what we do know (Stankiewicz 2009). Scholars aimed to rectify how little was known about ignorance, and to acknowledge the social and political centrality of ‘knowing what not to know’ (Taussig 1990) and how powerful the consequences of ignorance can be (Proctor 2008).

A key aim of ignorance studies has been to show that ignorance is not a passive state; it is not neutral or natural or simply an absence of knowledge, instead ignorance is just as socially constructed as knowledge (Reser and Smithson 1988; Sanabria 2015). This relationship between knowledge and ignorance has been extensively debated. McGoey (2012a) challenged the assumption that ignorance and knowledge are binary opposites and argued that ignorance is not the *absence* of knowledge, it is a *form* of knowledge (McGoey 2012b). In a similar vein, Ungar (2008 303) stated that “ignorance is inextricably tethered to knowledge” with no clear division between them and Bakonyi (2018) has demonstrated how knowledge and ignorance can be intertwined. Furthermore, McGoey (2020) has emphasized the importance of understanding the distinction between ‘unknowability’ and ‘ignorance.’ While unknowability refers to the inherent inability to know something, ignorance is more contextual and suggests the refusal or failure to know something. Given the breadth and depth of this work then, while Abbott (2010, 174) declared “a certain sociological ignorance of ignorance” over a decade ago, that seems to be less the case now.

Within ignorance studies, Linsey McGoey has been influential in developing work on strategic ignorance (2007; 2012a; 2012b; 2019; 2020). She defines it as “the structural ability to exploit the unknowns in an environment in order to gain more power or resources” (McGoey 2020, 198). ‘Structural’ here means that groups deliberately conceal knowledge, “making some societal ‘unknowns’ a collective achievement” (McGoey 2020, 198). The mobilization of ignorance for nefarious reasons has been commonly studied. Halliday (2018) describes it as something that can be cultivated, for Stankiewicz (2009) it can be wielded for political ends, and Kempner (2020) explains that while ignorance is unavoidably normal and an expected part of life, some types of ignorance are consciously produced for deleterious ends. McGoey (2012a) recognizes the dangerous mobilization of unknowns to command resources, deny liability and assert control but she also proposes the term ‘emancipative ignorance’ (2012b), which refers to using deliberate ambiguity as a weapon. This highlights how ignorance is often an outcome of cultural and political struggle (Schiebinger 2005: 320). Ignorance can have instrumental value (Gross and McGoey 2015), and that is for the powerless as well as the powerful.

Empirical research on ignorance has covered a range of topics including phone hacking (McGoey 2019), nuclear threats (Reser and Smithson 1988) and scientific research (Gaudet, 2013). Within health and medicine scholarship, ignorance has been studied in relation to the obesity ‘epidemic’ in France (Sanabria, 2016), medications (McGoey 2012a; 2007), HIV (Heimer 2012), the women’s health movement (Tuana 2006), and abortion (Schiebinger 2005; 2007). In her study of abortifacients in the West Indies, Londa Schiebinger (2005,342), found that while the peacock flower (used in the Caribbean by slaves and non-slaves to provoke an abortion) itself travelled to Europe, knowledge of its abortifacient properties did not. She ascribes this to “a kind of cultured apathy or cultivated indifference” that responded to contemporary mercantilist pronatalist policies celebrating children as “the wealth of the nation”, patterns of patronage and trade, and moral and professional imperatives. Knowledge of the abortifacient properties of plants was therefore slowly lost, particularly in Europe.

More recent work has furthered this scholarship on the relationship between ignorance and abortion. Jessica Marcotte (2016) shows that women have historically achieved a level of reproductive freedom not only through the creation of a system of knowledge to regulate fertility, but also through their participation in a system of ignorance that served to ‘sequester’ this knowledge. Marcotte explored how women historically concealed the use of ‘emmenagogues,’ usually plants which were used to stimulate menstruation, from men including husbands and physicians. Sally Sheldon (2018) and Irene Maffi (2022) have conducted research on how contemporary governments, policy makers and healthcare professionals have willfully cultivated ignorance around abortion pills in the Republic of Ireland and Tunisia, respectively. This paper furthers this work to explore strategic ignorance in abortion practices in Latin America in a ‘triangle of interactions’: the state, abortion providers and individuals who seek abortions. This multi-actor approach recognises that strategic ignorance is both individual and collective and is a scalar phenomenon (McGoey 2020), consisting of ‘varieties of ignorance’ (Abbott 2010). Through these actors we show how at multiple scales, the cultivation of ignorance can be advantageous as both a sword and a shield.

## The State: Denying The Problem Of Abortion

Abortion is one of the main bones of contention within the shifting historical configurations of reproductive governance globally (Morgan and Roberts 2012). In Latin America in particular, abortion has been a battleground where liberal and conservative struggles play out. This has resulted in a split on government’s attitudes towards the law and official practice regarding abortion. On the one hand there is a wave of liberalization as seen in Argentina and Colombia whereas others, including Honduras, El Salvador, Nicaragua and the Dominican Republic, have further restricted their abortion policies, narrowing the legal exceptions for an abortion, and even banning the practice without exceptions (Fernández Anderson 2020). Here we argue that these latter governments deploy forms of strategic ignorance as a central part of their governmental rationality. This allows them not only to evade responsibility for unsafe abortions, but also produces the ambiguity that allows for the reproduction of clandestinity.

In the twentieth century, Latin American presidents, predominantly military leaders, rewrote their countries’ abortion laws as part of a broader project of modernization and updating of their countries’ legislature. This meant that in the first third of the twentieth century, countries including Peru, Argentina, Uruguay and Chile changed their penal laws to legalise therapeutic abortions, but only when a pregnancy endangered a woman’s life (Necochea López 2014,57). The result was a series of restrictive abortion laws across the continent that only allowed abortions in exceptional circumstances. Any abortions that flouted these exceptional circumstances risked severe judicial punishment.

This criminalization of abortion does not stop it from happening, it just forces abortions into ‘clandestinity’. While such abortions are not by default unsafe, abortion seekers are left exposed “to the mercy of the elements” (Chaneton and Vacarezza 2011: 131), and any health risks disproportionately impact marginalized and racialized communities (Wurtz 2012; Singer 2019). It is also when people access healthcare in the case of complications that they can become exposed to judicial punishment (Salazar Vega 2019). Restrictive frameworks reflect “states of uncare”, meaning that care responsibilities are not only denied but those who attempt to access or facilitate abortions are actively punished (Duffy, Freeman and Rodríguez 2023, 610).

Yet governments choose to ‘turn a blind eye’ to these grim realities (McGoey 2012a). In part, this response is because abortion has become a so-called ‘battleground’ for its precise ability to mobilize political action, whether anti- or pro-abortion. Politicians in Latin America are often wary of being outspoken on the ‘costly political stance’ of abortion rights because they come under attack when they defend reproductive rights or attempt reform (Fernández Anderson 2016). Abortion is all too often framed by governments as a complex issue that there is simply not enough time to deal with amongst other problems. For instance, in Bolivia, Evo Morales’ Movimiento al Socialismo party put the legalisation of abortion aside claiming that there was not sufficient space to add another debate to the legislative agenda (Kimball 2020). Meanwhile in Mexico, President Andres Manuel Lopez Obrador has refused to legislate on abortion, stating that it is a decision for women, not one for him to dictate from government. Some leaders believe that they can claim neutrality by being opaque about their stance on abortion.

However, we argue that deliberately choosing-not-to-know serves a function. Governments refuse full knowledge of the magnitude of abortion practices and maintain ignorance in order to treat abortions as an anomaly. This ignorance is anything but accidental and feigning ignorance is an “expression of political will” itself (Halliday 2018, 937). Claiming that abortion is unknowable and ‘ungovernable’ is a form of governance in itself. The sheer number of clandestine abortions that take place in most Latin American countries is an inconvenient truth for governments. To uphold their structural strategic ignorance about the violent consequences of anti-abortion policies, many Latin American states deliberately avoid collecting abortion incidence data. Where abortions are clandestine and occur outside of any formal health system, gathering accurate data on their frequency is always going to be a difficult task. This is illustrated by the fact that Cuba, which has legalized abortion, is able to gather reliable data about abortion whereas countries without legal abortion, including Peru, rely on hospital admissions or targeted surveys which grossly underestimate abortion incidence (Paxman et al. 1993).

By preventing knowledge from emerging (McGoey 2020), states blinker themselves against the magnitude of the health burdens associated with clandestine abortion procedures. This manufactured unknowability serves to shape abortion as a specific problem of governance; one that pertains to the control and prosecution of deviant and ‘bad women’ rather than to the design of public health policies. This creates a moral cognitive dysfunction (Sullivan and Tuana 2007) that reinforces stigma and social sanction, further entrenching a “prevalence paradox” in which abortion exceptionality is assumed (Kumar, Hessini and Mitchell 2009). By choosing to remain deliberately ignorant, the state can uphold the fiction that abortion is an anomaly, which in turn justifies its criminalization. Remaining ignorant shifts the responsibility onto individuals and governments are not required to change any laws or provide access to healthcare. Ignorance becomes a productive asset through which the state evades responsibility and judicial and legal sanction become a legitimate governmental response (Stel 2016).

Given the difficulties around monitoring abortion, does the state wish to know when an illegal abortion, constituting a crime, has taken place? Prosecution is a very real threat, ruining lives and perniciously affecting those who have had a miscarriage if there is any suspicion that it may have been provoked. But, given the vast numbers of ‘illegal’ abortions taking place every year, prosecutions remain relatively low across Latin America (Fernández Anderson 2016). As Kimball (2020) shows in the context of Bolivia, there are several reasons for this fairly low number. It is difficult to prove that an abortion occurred as the effects of a medical abortion are indistinguishable from those of a miscarriage and in order to prosecute providers, they need to be caught in the act of performing an abortion or someone needs to provide a confession. Moreover, physicians have often been reluctant to report suspected abortions to the authorities because they were sympathetic, wanted to avoid the additional work, or didn’t want to make private affairs public and risk their reputation (Necochea López 2014).

Nevertheless, the fear of being caught, the interrogations by the authorities, being forced to pay bribes to the police, potentially losing jobs and the stigma faced with being publicly denounced or investigated are all part of the culture of fear created by the criminalization of abortion. There are police, doctors, and judges who actively seek to prosecute the procurement of abortion, and those targeted are disproportionately economically marginalized (Fernández Anderson 2016). In the last decade in Peru, 571 women have been taken to court accused of intentionally terminating their pregnancies and the number of complaints filed by the public prosecutor’s office is far higher (Salazar 2019). In Mexico, between 2007 and 2016, 3568 people were reported to the police under suspicion of procuring or providing an abortion (GIRE 2018). The fear that people feel when seeking an abortion is legitimate.

Given the hundreds of thousands of abortions that take place across Latin America every year, this culture of fear is clearly not preventing abortions from occurring. But rather than being the manifestation of a policy failure, we argue that this low number of prosecutions has a double utility. On the one hand, it helps maintain a culture of fear, where the violence of sanction is administered by the effects of state *absence* rather than its presence. The government’s *potenza* (Martin 2011, 195 in Stel 2016) is manifested in its potential abandonment of those who have a clandestine abortion and all its attendant risks. On the other hand, this low number of prosecution cases maintains the illusion of the efficacy of the bureaucratic apparatus of surveillance (Dedieu et al. 2015). More active prosecution would demonstrate that abortion is ubiquitous and highlight the inability of the state to prevent it. By keeping the number of prosecutions low and maintaining the illusion that surveillance mechanisms are effective, this form of strategic ignorance constructs public legitimacy and ensures the survival of its bureaucratic apparatus.

A second form of ignorance deployed by the state is the active production of ambiguity through the obfuscation of healthcare information and rights. In her study of Palestinian gatherings under Lebanese control, Stel (2016) introduces Dunn and Cons’ (2014,93) notion of ‘sensitive spaces’ instead of ‘spaces of exception’ to engage explicitly with ambiguity and uncertainty as core features of spaces where multiple modes of power and conflicting claims to sovereign control collide. Clandestinity is also determined by a pervasive uncertainty, unpredictability, and ambiguity where individuals seeking to have an abortion have to navigate their loss of political rights. The provision of ‘therapeutic abortions’ is an arena that illustrates how ambiguity and confusion can be exploited by the state in order to evade its responsibilities. Therapeutic abortions are technically legally available in most Latin American countries for very specific cases that tend to orbit around risk to the life of the gestating parent, foetal inviability and rape but that does not mean that access to abortion even where the criteria are filled is possible in practice. These technical criteria mean that on an international stage most Latin American states are following expectations that abortions will be legal for those in certain cases yet in reality these legal exceptions are very hard to access. They require a panel of doctors and/or judges, paperwork, knowledge that these exceptions exist, and time. With these exceptions placed within gestational limits, by the time someone has proven they qualify for one of the legal reasons it may well be too late for the procedure to legally be performed. Moreover, if legal access is granted, it may not be possible to find a clinic that will offer the procedure or a doctor willing to perform it (Pilecco et al. 2021). The result is that those who meet the criteria for a legal abortion are denied their right, forcing them to seek clandestine abortions.

In Latin American countries that have some provisos for legal abortion but no general right to an abortion, the state is creating confusion and misinformation about the legal position of abortion (Palomino et al. 2011). Those who work for the state may believe they are protecting women and ‘the unborn’ in a paternalistic way and that concealing the public from information about abortion is necessary to serve the public (McGoey 2007). This belief is misplaced as countries with strict abortion laws have higher mortality rates (Latt, Milner and Kavanagh 2019). Therefore, abortion “is often regulated in such a way that neither its safety nor women’s unburdened access to the procedure are guaranteed” (Kimball 2020, 236).

The state wields strategic ignorance by deliberately choosing to ‘not know’ the problem of abortion and by exploiting ambiguity and confusion within spaces of clandestinity. The state’s manufactured ignorance fulfills governmental logic and practice. By rendering abortion an anomaly, it justifies its criminalization and legitimates the efficacy of the bureaucratic apparatus. Remaining ignorant shifts the responsibility onto individuals, and governments are not required to change any laws or provide access to healthcare. This form of reproductive governance upheld by so many Latin American governments is a form of ‘wilful blindness’ (McGoey 2019); abortions that occur beyond state regulatory systems officially do not exist (Kimball 2020). At the same time, these forms of strategic ignorance produce an ironic outcome: they render the state incapable of understanding the world it has helped to create (Mills 2007). Their unknowability of clandestine abortions is what has allowed a flourishing of alternative methods to access an abortion. Hence, they paradoxically provide the conditions where clandestine abortions can be sought and kept secret. We now turn to the forms of strategic ignorance as wielded by ‘the powerless’, providers and individuals seeking abortions, which respond to the tactics of the authorities in the cracks of these marginal and clandestine spaces.

## Providers: Support In The Shadows

Performing an abortion typically carries a severe prison sentence across the continent. As a result, providers have to maintain secrecy and privacy as a necessary protection against prosecution. As one Peruvian abortion provider put it, “one of the strategies - and it angers me to say it - has been to keep quiet.” Yet, at the same time, abortion providers face a fundamental problem: in order to be effective in expanding access to abortion *they have to be known*. Therefore, their secret practice has to be both publicly known *and* unknown. To uphold the paradoxical nature of their practice, providers create structural strategic ignorance around their work through two interrelated strategies: concealment and erasure. The deployment of these strategies is based on a detailed knowledge of the law and the simultaneous circulation of knowledge and ignorance around abortion practices. Providers manufacture a partial ignorance, sculpting the *chiaroscuros* of what is made visible and what remains in the shadows.

There is a wide spectrum of abortion providers across Latin America. Here we focus on those who directly provide abortions or information about abortions, whether surgical or medication (the taking of medications) across three main groups. First, there are private or public sector healthcare professionals who perform surgical or medication abortions, usually in addition to their work in family planning services or obstetrics-gynaecology. Second, there are *acompañantes* [accompaniers]: people who support others with medication abortions in-person or virtually by phone or text and who often give emotional as well as practical support. Third, there are information providers who disseminate information about legal abortions, abortion rights, places to procure a surgical abortion and/or how to access and self-manage a medication abortion. While there are clear differences between these groups, we discuss them together here due to the blurring between them. For example, the Socorristas en Red, an accompaniment network in Argentina, explicitly work with health professionals to promote empathetic and anti-discriminatory abortion care on their part (Zurbriggen, Keefe-Oates and Gerdts 2018). In addition, there is a mixing of who is a ‘healthcare professional’ and who is an ‘activist’. There are health professionals who work as insider activists from within the health system and activists who build networks with healthcare providers (Fernández Vázquez and Szwarc 2018). For Sutton and Vacarezza (2021,11), “…radical abortion activism is not necessarily about always working against the state or completely outside of it.”

While many countries in Latin America have legislation that prohibits most abortions, there are opportunities to create space for abortions or information about them at the margins of the law (Ruibal and Fernández Anderson 2020). Laws can be interpreted in ways that allow for direct action strategies such as providing information or abortions “through a broad understanding of the health exception” (ibid, 705). Abortion providers work in an ‘alegal’ grey zone while resisting state control of abortion to provide care where that has been denied by the state (Singer 2022). It is precisely the detailed knowledge of the law that allows providers and activists to manufacture *partial* ignorance: they provide and circulate information among people who may need it, while also manufacturing the state’s ignorance about their practices. This careful manoeuvring of transparency and opacity, closure and disclosure, allows them to construct their practice as a ‘public secret’. For Burke (2023, 16), secrecy is characterized by keeping a small group ‘in the know’ and a large group ‘out of the loop’. Providers dissect and layer the public sphere, carefully selecting what type of information is made visible, what is kept in the shadows, and who gets to be in the know. Providers make sure that anti-abortion healthcare professionals and the police are unable to ‘know’ that an abortion has taken place, thereby shielding themselves and abortion seekers from the law. Even when the work that abortion providers do is an ‘open secret,’ providers manufacture formal denial of their practice to ensure that prosecutors would struggle to put together a convincing prosecution against them.

In this alegal grey zone, two interrelated strategies allow providers to publicly circulate sequestered knowledge while avoiding harm from authorities: concealment and erasure. Concealment strategies can be constructed through the careful use of language and the performance of professed ignorance in front of authorities. Providers must be very careful with the language they use to tell women how to abort. In communication about abortions between abortion providers (particularly information providers) and those seeking an abortion, an indirect way of providing information or a creative language of secrecy and codes is often deployed.

Misoprostol is a medication that has the secondary effect of provoking abortions and is accessible due to its official use for treating stomach ulcers. While misoprostol is most effective at ending pregnancies when taken together with another pill, mifepristone, misoprostol alone has been shown to have efficacy rates of 88-93% in clinical studies (Cohen et al. 2005) and has transformed access to safe abortions (Freeman 2020; Calkin and Freeman 2019). Providers may draw on other national, international, or public health legal frameworks to validate their ‘information provision.’ For example, many activists use the World Health Organization’s classification of misoprostol as an ‘essential medicine’ to justify their work, as *just* providing information is not illegal, and the global mobility of abortion pills challenges national legal frameworks (Calkin 2021). Their hyper-awareness of the flex in the law means they know what can and cannot be said and so information is often given exclusively using the third person. As one *acompañante* in Peru explained,

at least in writing, we don’t give any more information than is necessary, but rather as ‘you can read this guide,’ or ‘the WHO recommends such a thing,’ we would never say ‘I recommend such a thing,’ or ‘I tell you this’ or ‘you should do this’ if we don’t already know them. We make it a little more indirect.

Another described the importance of being vigilant with using the third person because you never know if you are being covertly recorded.

A creative language of secrecy and codes enables providers to feign ignorance as euphemisms create space for denial (Thiel 2015). Providers use a range of nicknames to avoid directly using terms such as ‘abortion’ or ‘misoprostol’ to avoid detection. One abortion Facebook group in Mexico requires users to confirm that they will never use the word ‘misoprostol’ in the group if they wish to join. In another Peruvian abortion Facebook group people use playful codewords so that an onlooker could not prove these terms meant ‘misoprostol.’ Given recent attacks on abortion providers in Latin America (Drovetta, Freeman and Rúa 2023), we have chosen not to include the specific names, but they often revolve around food or Catholic imagery. This creates plausible deniability and allows discussions around abortion access to remain undetected.

Another strategy of concealment is the performance of professing ignorance in front of authorities. Providers must protect themselves from prosecution if they are to continue their abortion care, and they train themselves to know when and how to deny their practice. But this doesn’t end with themselves: the training of women is a key part of manufacturing ignorance on behalf of authorities. One provider who performed abortions at a clinic in Peru explained that empowering the women they support is an important part of protection. It is imperative that these women understand what they are doing and are on the side of the providers so that if they are raided and questioned by the police that they will not say that they are at the clinic for an abortion. Providers check that patients are aware of the importance of this as a condition to receive care.

The providers also protect themselves through the erasure of compromising evidence or the falsification of evidence, common strategies to produce or maintain ignorance (Proctor and Schiebinger 2008). The lack of evidence makes prosecution for practicing an abortion much harder and allows the provider to claim ignorance about any illegal abortions having occurred. For example, one abortion provider in Peru asks people to delete any written evidence of their conversations on their mobile phones, others will never give out their real names or meet people they did not already know, or will use ‘burner phones’. In this way, genuine ignorance or plausible deniability can be created.

Abortion providers are also very careful with how they record any abortions that have been performed. For example, they use anonymized spreadsheets so that no procedures can be linked to individuals. One provider interviewee explained that during police raids all paperwork and computers have been seized so this anonymity is of utmost importance. We feel the need to be cautious here to protect our interviewees but there are detailed and rigorous forms of creating paperwork that provide an evidence trail for why individuals were visiting a certain clinic and why they may have required a gynaecological procedure. This is particularly necessary if during a raid the police find a patient mid-procedure, paperwork can provide cover for the provider and the patient. Layers of paperwork including consent forms and medical images are kept to deliberately conceal the abortions and to promote ignorance on behalf of the authorities. Heimer (2012) calls this obfuscation ‘sequestered knowledge’, as those who do not know what they are looking for will be overwhelmed with bewildering paperwork.

Those involved in providing access to abortion simultaneously wield knowledge and ignorance in order to work at the margins of the law (Bakonyi 2018). Through this, providers show how the border between knowledge and ignorance is continually negotiated (Gross and McGoey 2015). They embrace the paradoxical nature of their practice as a ‘public secret’, strategizing ways to circulate information for some and create ignorance on the part of others.

They rely on their detailed knowledge of the law and prosecution tactics around abortion to uphold structural strategic ignorance about the abortions they are providing as a way to protect their own practices but also to protect the individuals seeking abortions.

## Individuals: Concealing The Procedure

“In Peru it has always been illegal so in that sense we have always had to look after the issue ourselves”

Maintaining secrecy and privacy has played a fundamental role in creating pathways to abortion access in restricted settings not only for providers and activists but also for individuals seeking an abortion themselves. Unlike the former, individuals do not have to deal with the oxymoronic nature of the ‘public secret’ of their practice, instead they generally keep it within their close networks or to themselves. Here we consider the strategies employed by individuals to conceal their own abortions from three groups: members of their social networks who could obstruct their access to abortion, those selling them the abortion pills, and the medical staff or authorities they encounter if they experience complications. Concealment is guaranteed not only by staying silent, but also by mobilizing deliberate ambiguity and professed ignorance in order to create a ‘theatre of ignorance’ -a carefully arranged play of transparency and opacity, closure and disclosure- that allows individuals to overcome barriers to abortion access. Shepard (2000,115) explains that as part of the ‘double discourse system,’ such strategies are common and “constitute an escape valve that expands citizens’ sexual and reproductive choices.” This resonates with Marcotte’s (2016) argument that ‘culturally-induced’ ignorance has allowed women to broaden their reproductive freedoms. Through this theatre of ignorance women build invaluable epistemological camouflage in their practice of fertility control (Marcotte 2016). In the face of repressive reproductive governance, they are reclaiming the agency of the “reproductive subject” (Morgan and Roberts 2012). While these strategies show the emancipatory potential of ignorance (McGoey 2012b, McGoey 2019), it is important to note that while secrecy, privacy and concealment can protect individuals, it also comes with risks.

Individuals can experience greater anxiety, stress, and are at greater risk of suffering health complications when seeking abortions without support (Casas and Vivaldi 2014; Dides, et al. 2015; Ramos et al 2014). Family and friends can be a crucial source of economic and emotional support for individuals, even if they do not fully agree with their decision (Duffy, Freeman and Rodríguez 2023, Lafaurie et al. 2005), which can mediate the effects of abortion stigma (Kumar et al. 2009). However, they can also be a source of anxiety, exacerbate feelings of shame and guilt, or may obstruct abortion access. Individuals therefore enact ‘strategic ploys’ (Proctor 2008,3) to conceal their pregnancy, their search for an abortion, and the interruption itself. The story of a Peruvian woman we interviewed illustrates how theatres of ignorance can allow for support from some while avoiding harm from others. This woman asked for help from a close friend to carry out a self-managed abortion. However, when the procedure was not effective, she decided that silence was no longer an option and felt obliged to tell her partner, even though she was afraid he could physically abuse her. So, with the help of her friend and a health provider from the local health centre, she devised a plan where only a partial truth was revealed to him. When she and her partner went to the local health centre to be examined, the health provider convinced them that the pregnancy had a high risk of birth defects and that termination would be the best option. The provider even shared the contact of a private clinic that performs clandestine surgical abortions. In this way, she created a theatre of ignorance where her pregnancy was revealed but her intentions remained concealed; fabricating the abortion as a medical recommendation protects her from blame and abuse from her partner and allows her to reclaim authority over her own decision.

When self-managing an abortion, individuals often need to procure their own abortion pills, predominantly misoprostol in Latin America, which are available from pharmacies (usually with a prescription) or on the black market. Misoprostol was originally designed as a stomach ulcer medication and that means it is accessible (even if not legally) for abortions (Freeman 2020). This ‘double life’ of misoprostol (De Zordo 2016) means that those buying the pills from a pharmacy need to avoid raising suspicion that they will use them for an abortion through deliberate ambiguity or find a pharmacy that is willing to sell them for an abortion. In our research we found that prospective buyers were able to give partial or limited information about why they were buying misoprostol. This included sending someone else who did not fit the stereotype of the ‘aborting woman’ in to buy the pills. One *acompañante* in Mexico would buy misoprostol on behalf of younger women because at her age pharmacy staff would presume she was post-menopausal and therefore could not be seeking an abortion. Another *acompañante* explained that “getting misoprostol for women is more difficult than for men. We have got, for example, a male friend of ours to buy us the pills.” An *acompañante* group who are located in an area of Peru where it was difficult to buy misoprostol from pharmacies found that if they sent men they were more likely to be successful and also to be charged a lower price. The ‘double life’ of misoprostol with its treatment for stomach ulcers as well as for abortion means they could send these *viejitos ulcerosos* [ulcerous old men] to buy the pills. One *acompañante* interviewee explained that the pharmacy staff are suspicious of these men but that there is nothing they can do about it because there’s no proof they are buying them for abortions. Hence, by exploiting deliberate ambiguity with regards to their motives, they guarantee access to misoprostol.

For this buying of misoprostol from pharmacies to work, strategic ignorance on the part of the pharmacy staff is required. Pharmacy staff play an important role in whether access to the pills is possible and they may need to be willing to take part in a theatre of ignorance to ‘look the other way’ when they have reason to suspect the medication is being bought for an abortion. Some pharmacy staff do not ask for a doctor’s prescription even in jurisdictions where this is required by law whereas others are willing to accept suspect prescriptions. For example, one *acompañante* explained how she had been able to buy misoprostol using the same prescription for two years even though it was clearly old. The pharmacy staff uphold the public secret of knowing what not to know (Taussig 1999) by not overtly articulating the sale of misoprostol for abortions. This is a widespread practice in Latin America with one *acompañante* explaining, “[misoprostol] is hardly ever used for what it is intended for - ulcers - everyone who goes to the pharmacy and asks for misoprostol is getting them to have an abortion, especially when they ask for twelve pills [the WHO recommended regimen].” This is increasingly the case as more effective, cheaper ulcer medications are preferred for the treatment of gastrointestinal issues. Notably, pharmacy staff are not always altruistically providing much-needed healthcare services in a state that denies them, they often charge highly inflated prices of up to five times higher than when sold with a prescription. However, strategic ignorance by pharmacy staff does allow for the possibility of self-managed abortions with misoprostol.

When abortions are performed correctly they are almost always safe and effective but complications can occur in rare cases. If someone experiences severe bleeding they may be haemorrhaging and require intervention. The abortion pill misoprostol can be taken by dissolving the pills between the gum and the cheek, under the tongue, or vaginally and there is no blood or urine test that can identify it. This means the effects are indistinguishable from that of a miscarriage unless residue from the pills is discovered in the vagina. However, it is not uncommon for patients to admit that they have taken misoprostol in the hope that will aid their treatment. In reality, the treatment is identical and confessing to the use of misoprostol in settings where abortion is legally restricted may spark the chain of prosecution that could end in imprisonment. By knowing how to administer the pills (i.e. not vaginally) and by knowing what to say to medical staff and the police it is possible to evade the political consequences of potential residues.

Misoprostol users are trained by *acompañantes* and providers on what to say and how to ‘perform’ if they do have to go to hospital because of excessive bleeding. This creates a ‘theatre of ignorance’ through knowing what to say when they arrive at a hospital with complications arising from a clandestine abortion and whether to act devastated at this spontaneous miscarriage or shocked to find out they were pregnant at all. Even if hospital staff suspect the abortion may have been provoked, they are unable to prove it and are therefore ‘off the hook’ from reporting a suspected abortion, just as pharmacy staff can look the other way if they have not been given any reason to suspect misoprostol is being bought for its abortifacient properties. Individuals are trained by providers with ‘scripts’ to explain they “started bleeding out of nowhere” and what symptoms, feelings, and timescale to report. Individuals are thus prepared to invoke professed ignorance and perform the role of someone who has experienced a miscarriage and construct ignorance on the part of the medical staff who treat them. For Necochea López (2014,77) this is “a form of power at work here, a subaltern kind of power that denies access to the “truth” or at least to a confession.”

The strategic use of ignorance by abortion seekers in a context where the practice is considered illegal shows the emancipative power of ignorance (McGoey 2012b). Individuals set up a theatre of ignorance, where transparency and opacity regarding their condition and motives are carefully displayed depending on the different audiences they (are forced to or choose to) encounter in ways that allow them to access abortion while avoiding harm. This play of closure and disclosure is created by staying silent, invoking professed ignorance and exploiting deliberate ambiguity. In this ‘theatre of ignorance’ truth does not necessarily need to be revealed nor denied. For instance, deliberate ambiguity is used in the buying of abortion pills as the older women or men buying the pills are not explicitly claiming to be buying them for stomach ulcers, but they are strategically creating the impression that they are. In other words, the strategic deployment of ignorance helps them to uphold the secrecy and privacy needed to navigate a landscape of clandestinity where risks for women are manyfold.

However, we should not lose sight that these strategies are but a vital response to a punitive state. Those seeking an abortion are too often *forced* into secrecy, clandestinity and erasure. This is not out of choice but is necessary because of the institutions of repression that make other forms of resistance challenging (Scott 1985). The strategic use of ignorance can be understood as an outcome of political struggle that is also deployed by ‘the weak’, individuals and providers, in ways that do not constitute a revolution or collective defiance but are much more ‘ordinary weapons’ (Scott 1985). Such forms of ‘emancipative ignorance’ (McGoey 2012b) become tools of survival in contexts of reproductive governance that punish people for managing their own fertility and reproductive lives. The problem is that, as Stel argues (2016,1415) “they work around, or even with, the foundation of domination rather than shaking them”. As we explored earlier, strategic ignorance is a central part of governmental rationality and practice, allowing the state to evade responsibility and maintain clandestinity. For this reason, we argue that silence, secrecy and concealment simultaneously protect and endanger individuals and providers. Hence, it is understandable why for the Peruvian provider mentioned above, using silence as a strategy angered her, because she is fully aware that while it means abortions can be practiced, it exposes her and her patients to multiple risks. As Shepard (2000,115) argues, these strategies may be necessary escape valves “but because they are makeshift, illegal, or unofficial, neither availability, safety (in the case of services), nor protections of basic rights are guaranteed.” Hence, for women who venture on journeys to terminate their pregnancies, the use of strategic ignorance blurs the line between resistance and suicide (Mbembe 2019), as they have been previously forced by the state to live at the ‘edge of life’ (Rodríguez 2020). Moreover, the use of strategic ignorance by ‘the weak’ allows the state to continue evading responsibility, upholding the current landscape of clandestinity.

## Conclusion

State departments refusing to collect abortion incidence data. Healthcare systems that fail to provide access to abortions under their own narrow ‘exceptions’. A volunteer on a phone line speaking in the third person when they just want to comfort the caller. A gynaecologist writing on their spreadsheet that their patient ‘had a coil fitted’. A man who goes into the pharmacy claiming to be suffering from stomach ulcers. People practicing what to say if a doctor asks them when they started bleeding. These strategies may not seem to have much in common with one another but we argue it is the combination of these strategies together that creates strategic ignorance and upholds the current landscape of clandestine abortion in Latin America. In this paper we have made an empirical contribution to abortion scholarship by setting out the strategies that conceal abortion in Latin America and a theoretical contribution to ignorance studies through exploring how strategic ignorance is enacted ‘top-down’ and ‘bottom-up’ in individual and collective ways.

Strategic ignorance maintains abortion clandestinity in Latin America and makes the possibility of access to clandestine abortions a reality. Across the ‘triangle of interactions’ (Reagan 2022:1) we presented here, from the state to providers, and individuals, there is power in ‘knowing what not to know’ (Taussig 1990). When wielded by the state, strategic ignorance reproduces the status quo of the criminalization of abortion but when wielded by abortion providers and individuals it creates the conditions for abortions to be procured without prosecution. Many states choose to remain deliberately ignorant, providers work under a screen of secrecy and strategically work at the margins of the law, and individuals strategize to keep pharmacy and medical staff ignorant. Through these strategies, abortion becomes a public secret, one that is known but not always known to be known.

There are clear winners and losers in this story of ignorance. Elite political groups maintain their power and significant funding while reproductive healthcare remains restricted and clandestine abortions continue, with racialized and classed effects. If strategic ignorance is “the structural ability to exploit the unknowns in an environment in order to gain more power or resources” (McGoey 2020, 198) then it is cultivated by the powerful state as well as the less powerful providers and individuals for their own ends. The ‘emancipative’ strategic ignorance (McGoey 2012b) performed by providers and individuals is a necessary form of resistance in contexts where abortions are not legally accessible. Ignorance and secrecy saves lives, protects livelihoods and shields people from public shaming and prosecution. The strategies outlined here create ambiguity around abortion. They mean that abortions can be accessed, and increasingly safely, without being sanctioned by the state. At the same time, it is important not to overstate the power or organisational capabilities of the state. There is not always a highly orchestrated system of ignorance masterminded by a cabal of anti-abortion elites. Decisions to ignore information or neglect to know it may be deliberate or may be inadvertent and the line between them is not always clear (Proctor 2008). However, while strategic ignorance is utilized by the powerless to claim bodily autonomy it also upholds clandestinity. This further reinforces misconceptions and misinformation about abortion, reproduces stigma and keeps abortion as a shadowy, secret phenomenon.

Yet clandestinity is not the only option. As recent developments in Argentina, Mexico, and Colombia have shown, states that have refused to create legal pathways to abortion access can change. Legislation should be viewed sceptically as without full decriminalization and the provision of broad abortion options for all, abortions will remain inaccessible and stigmatized. As the abortion activists introduced in this paper show, safe, empathetic, and supported abortions are possible beyond the state. Rather than accepting crumbs from the state, we must fight for free and autonomous reproductive justice for all.

## Data Availability

Given the nature of our data, interview transcripts are not publicly available. Please contact the authors to discuss data access further.

## References

[R1] Abbott Andrew (2010). Varieties of Ignorance. American Sociologist.

[R2] Bakonyi Jutta (2018). Seeing like bureaucracies: Rearranging knowledge and ignorance in Somalia. International Political Sociology.

[R3] Bearak Jonathan, Popinchalk Anna, Ganatra Bela, Moller Ann-Beth, Tunçalp Özge, Beavin Cynthia, Kwok Lorraine, Alkema Leontine (2020). Unintended pregnancy and abortion by income, region, and the legal status of abortion: estimates from a comprehensive model for 1990-2019. The Lancet Global Health.

[R4] Billings Deborah (2004). Misoprostol alone for early medical abortion in a Latin American clinic setting. Reproductive Health Matters.

[R5] Calkin Sydney (2021). Transnational abortion pill flows and the political geography of abortion in Ireland. Territory, Politics, Governance.

[R6] Calkin Sydney, Freeman Cordelia (2019). Trails and technology: social and cultural geographies of abortion access. Social & Cultural Geography.

[R7] Casas Lidia, Vivaldi Lieta (2014). Abortion in Chile: the practice under a restrictive regime. Reproductive health matters.

[R8] Céspedes Pamela, Renteria EdanaR, Pinto JosephA (2020). The hidden impact of the clandestine abortion in Peru. Journal of Public Health Policy.

[R9] Chaneton July, Vacarezza Nayla (2011). La intemperie y lo intempestivo: experiencias del aborto voluntario en el relato de mujeresy varones.

[R10] Cohen Jessica, Ortiz Olivia, Llaguno SilviaElena, Goodyear Lorelei, Billings Deborah, Martinez Imelda (2005). Reaching women with instructions on misoprostol use in a Latin American country. Reproductive Health Matters.

[R11] De Zordo Silvia (2016). The biomedicalisation of illegal abortion: the double life of misoprostol in Brazil. História, Ciências, Saúde-Manguinhos.

[R12] Dides Claudia, Fernández Constanza, Peltier Gwendoline (2015). Aborto en Chile: cifras y testimonios que respaldan la exigencia de la legalización del aborto por tres causales. Nomadías.

[R13] Drovetta RI, Freeman C, Rúa A (2023). Self-Care for Abortion Activists and Providers: Lessons of Law and Risk from Argentina. BMJ Sexual & Reproductive Health.

[R14] Duarte NandaIseleGallas, Da Silva VeraLuciaMarques, Pinto LianaWernersbach (2020). A amiga que já abortou: um olhar sobre experiências partilhadas em uma comunidade virtual. Ciência & Saúde Coletiva.

[R15] Dunn E, Cons J (2014). Aleatory sovereignty and the rule of sensitive spaces. Antipode.

[R16] Duffy DeirdreNiamh, Freeman Cordelia, Castañeda SandraRodríguez (2023). Beyond the state: abortion care activism in Peru. Signs.

[R17] Fernández Anderson Cora (2020). Fighting for abortion rights in Latin America.

[R18] Fernández Anderson Cora (2016). Reproductive Inequalities: As Latin America’s pink tide recedes, the struggle for reproductive health reform continues. NACLA Report on the Americas.

[R19] Vázquez Fernández Sandra, Szwarc Lucila (2018). Aborto medicamentoso. Transferencias militantes y transnacionalización de saberes en Argentina y America Latina. RevIISE-Revista de Ciencias Socialesy Humanas.

[R20] Ferrando Delicia (2006). El aborto clandestino en el Peru: Hechos y cifras.

[R21] Freeman Cordelia (2020). Viapolitics and the emancipatory possibilities of abortion mobilities. Mobilities.

[R22] Gaudet Joanne (2013). It takes two to tango: knowledge mobilization and ignorance mobilization in science research and innovation. Prometheus.

[R23] GIRE (2018). Maternidad o Castigo La criminalización del aborto en México.

[R24] Gross Matthias, McGoey Linsey (2015). Routledge International Handbook of Ignorance Studies.

[R25] Halliday TerenceC (2018). Plausible folk theories: Throwing veils of plausibility over zones of ignorance in global governance. The British Journal of Sociology.

[R26] Heimer CarolA (2012). Inert facts and the illusion of knowledge: Strategic uses of ignorance in HIV clinics. Economy and Society.

[R27] Herrera García Alfonso (2022). Jurisprudencia constitutional de la Suprema Corte de Justicia de México en 2021. Anuario Iberoamericano de Justicia Constitucional.

[R28] Ismail Salwa (2006). Political Life in Cairo’s New Quarters: Encountering the Everyday State.

[R29] Kempner Joanna (2020). Post-Truth and the Production of Ignorance. Sociological Forum.

[R30] Kimball NatalieL (2020). An Open Secret: The History of Unwanted Pregnancy and Abortion in Modern Bolivia.

[R31] Kumar Anuradha, Hessini Laura, Mitchell Ellen (2009). Conceptualizing abortion stigma. Culture, Health, and Sexuality. An International Journal for Research, Intervention and Care.

[R32] Lafaurie MariaMercedes, Troncoso Erika, Billings Deborah, Chávez Susana, Maira Gloria, Martinez Imelda, Mora Margoth, Ortiz Olivia (2005). Las experiencias de las mujeres en México, Colombia, Ecuador y Perú.

[R33] Latt SuMon, Milner Allison, Kavanagh Anne (2019). Abortion laws reform may reduce maternal mortality: an ecological study in 162 countries. BMC Women’s Health.

[R34] Maffi Irene (2022). The production of ignorance about medication abortion in Tunisia: between state policies, medical opposition, patriarchal logics and Islamic revival. Reproductive Biomedicine & Society Online.

[R35] Marcotte Jessica (2016). The agnotology of abortion: A history of ignorance about women’s knowledge of fertility control. Outskirts: Feminisms Along the Edge.

[R36] Mbembe Achille (2019). Necropolitics.

[R37] McGoey Linsey (2007). On the Will to Ignorance in Bureaucracy. Economy and Society.

[R38] McGoey Linsey (2012a). The logic of strategic ignorance. The British Journal of Sociology.

[R39] McGoey Linsey (2012b). Strategic unknowns: Towards a sociology of ignorance. Economy and Society.

[R40] McGoey Linsey (2019). The Unknowers: How Strategic Ignorance Rules the World.

[R41] McGoey Linsey (2020). Micro-Ignorance and Macro-Ignorance in the Social Sciences. Social Research: An International Quarterly.

[R42] McReynolds-Pérez Julia (2017). No doctors required: Lay activist expertise and pharmaceutical abortion in Argentina. Signs: Journal of Women in Culture and Society.

[R43] Mills Charles, Sullivan S, Tuana N (2007). Race and Epistemologies of Ignorance.

[R44] Morgan LynnM (2019). Reproductive governance, redux. Medical Anthropology.

[R45] Morgan LynnM, Roberts Elizabeth (2012). Reproductive governance in Latin America. Anthropology & Medicine.

[R46] Necochea López Raúl (2014). A History of Family Planning in Twentieth-century Peru.

[R47] Palomino Nancy, Padilla Miguel Ramos, Talledo Brigitte Davey, Mazuelos Christian Guzman, Carda Jessica, Bayer AngelaM (2011). The social constructions of unwanted pregnancy and abortion in Lima, Peru. Global Public Health.

[R48] Paxman JohnM, Rizo Alberto, Brown Laura, Benson Janie (1993). The clandestine epidemic: the practice of unsafe abortion in Latin America. Studies in Family Planning.

[R49] Pilecco FláviaBulegon, Mccallum CeciliaAnne, De Almeida MariaDaConceiçãoChagas, Alves FláviaJôseOliveira, Rocha AlineDosSantos, Ortelan Naiá, Gabrielli Lígia, De Souza Menezes GreiceMaria (2021). Abortion and the COVID-19 pandemic: insights for Latin America. Cadernos de Saúde Pública.

[R50] Proctor Robert, Schiebinger Londa (2008). Agnotology: The Making and Unmaking of Ignorance.

[R51] Proctor Robert (2006). ‘Everyone knew but no one had proof’: Tobacco industry use of medical history expertise in US courts, 1990 2002. Tobacco Control.

[R52] Proctor Robert, Proctor Robert, Schiebinger Londa (2008). Agnotology: The Making and Unmaking of Ignorance.

[R53] Ramos Silvina, Romero Mariana, Aizenberg Lila (2014). Women’s experiences with the use of medical abortion in a legally restricted context: the case of Argentina. Reproductive Health Matters.

[R54] Reagan LeslieJ (2022). When Abortion Was a Crime.

[R55] Reser JosephP, Smithson MichaelJ (1988). When ignorance is adaptive: Not knowing about the nuclear threat. Knowledge in Society.

[R56] Rodríguez Sandra (2020). The psychopolitics of abortion: women’s experiences of abortion in Peru.

[R57] Roth Cassia (2020). A Miscarriage of Justice: Women’s Reproductive Lives and the Law in Early Twentieth-century Brazil.

[R58] Ruibal Alba, Anderson CoraFernández (2020). Legal obstacles and social change: strategies of the abortion rights movement in Argentina. Politics, Groups, and Identities.

[R59] Salazar Elizabeth (2019). Abortar en Peru: cuando víctima y familiares son llevados a cárcel.

[R60] Sanabria Emilia (2016). Circulating ignorance: Complexity and agnogenesis in the obesity epidemic. Cultural Anthropology.

[R61] Schiebinger Londa (2005). Agnotology and exotic abortifacients: The cultural production of ignorance in the eighteenth-century Atlantic world. Proceedings of the American Philosophical Society.

[R62] Schiebinger Londa (2007). Plants and Empire.

[R63] Scott James C (1985). Weapons of the Weak: Everyday Forms of Peasant Resistance.

[R64] Sheldon Sally (2018). Empowerment and privacy? Home use of abortion pills in the Republic of Ireland. Signs.

[R65] Shepard Bonnie (2000). The ‘double discourse’ on sexual and reproductive rights in Latin America. Health and Human Rights.

[R66] Singer ElyseOna (2022). Lawful Sins: Abortion Rights and Reproductive Governance in Mexico.

[R67] Singer ElyseOna (2019). Abortion exile: navigating Mexico’s fractured abortion landscape. Culture, Health & Sexuality.

[R68] Stankiewicz Piotr (2009). The role of risks and uncertainties in technological conflicts: Three strategies of constructing ignorance. Innovation.

[R69] Stel Nora (2016). The agnotology of eviction in South Lebanon’s Palestinian Gatherings: How institutional ambiguity and deliberate ignorance shape sensitive spaces. Antipode.

[R70] Sullivan Shannon, Tuana Nancy (2007). Race and Epistemologies of Ignorance.

[R71] Sutton Barbara (2017). Zonas de clandestinidad y nuda vida: Mujeres, cuerpo y aborto. Revista Estudos Feministas.

[R72] Sutton Barbara, Vacarezza NaylaLuz, Sutton Barbara, Vacarezza NaylaLuz (2021). Abortion and Democracy.

[R73] Taussig Michael (1999). Defacement: Public Secrecy and the Labor of the Negative.

[R74] Thiel Darren, Gross Matthias, Mcgoey Linsey (2015). International Handbook of Ignorance Studies.

[R75] Tuana Nancy (2006). The speculum of ignorance: The women’s health movement and epistemologies of ignorance. Hypatia.

[R76] Ungar Sheldon (2008). Ignorance as an under-identified social problem. The British Journal of Sociology.

[R77] Wurtz H (2012). Indigenous women of Latin America: unintended pregnancy, unsafe abortion, and reproductive health outcomes. Pimatisiwin.

[R78] Zurbriggen Ruth, Keefe-Oates Brianna, Gerdts Caitlin (2018). Accompaniment of second-trimester abortions: the model of the feminist Socorrista network of Argentina. Contraception.

